# Features of Two New Proteins with OmpA-Like Domains Identified in the Genome Sequences of *Leptospira interrogans*

**DOI:** 10.1371/journal.pone.0122762

**Published:** 2015-04-07

**Authors:** Aline F. Teixeira, Zenaide M. de Morais, Karin Kirchgatter, Eliete C. Romero, Silvio A. Vasconcellos, Ana Lucia T. O. Nascimento

**Affiliations:** 1 Centro de Biotecnologia, Instituto Butantan, Sao Paulo, SP, Brazil; 2 Programa de Pós-Graduação Interunidades em Biotecnologia,Instituto de Ciencias Biomedicas, Universidade de Sao Paulo, Sao Paulo, SP, Brazil; 3 Laboratório de Zoonoses Bacterianas, Faculdade de Medicina Veterinária e Zootecnia, Universidade de Sao Paulo, Sao Paulo, SP, Brazil; 4 Nucleo de Estudos em Malária, Superintendência de Controle de Endemias - Instituto de Medicina Tropical, Universidade de Sao Paulo, Sao Paulo, SP, Brazil; 5 Centro de Bacteriologia, Instituto Adolfo Lutz, Sao Paulo, Brazil; Federal University of Pelotas, BRAZIL

## Abstract

Leptospirosis is an acute febrile disease caused by pathogenic spirochetes of the genus *Leptospira*. It is considered an important re-emerging infectious disease that affects humans worldwide. The knowledge about the mechanisms by which pathogenic leptospires invade and colonize the host remains limited since very few virulence factors contributing to the pathogenesis of the disease have been identified. Here, we report the identification and characterization of two new leptospiral proteins with OmpA-like domains. The recombinant proteins, which exhibit extracellular matrix-binding properties, are called Lsa46 - LIC13479 and Lsa77 - LIC10050 (Leptospiral surface adhesins of 46 and 77 kDa, respectively). Attachment of Lsa46 and Lsa77 to laminin was specific, dose dependent and saturable, with K*_D_* values of 24.3 ± 17.0 and 53.0 ± 17.5 nM, respectively. Lsa46 and Lsa77 also bind plasma fibronectin, and both adhesins are plasminogen (PLG)-interacting proteins, capable of generating plasmin (PLA) and as such, increase the proteolytic ability of leptospires. The proteins corresponding to Lsa46 and Lsa77 are present in virulent *L*. *interrogans* L1-130 and in saprophyte *L*. *biflexa* Patoc 1 strains, as detected by immunofluorescence. The adhesins are recognized by human leptospirosis serum samples at the onset and convalescent phases of the disease, suggesting that they are expressed during infection. Taken together, our data could offer valuable information to the understanding of leptospiral pathogenesis.

## Introduction

Leptospirosis, a worldwide zoonotic infection, is an important human and veterinary health problem. The etiologic agent of the disease is pathogenic *Leptospira*. Leptospirosis has greater incidence in tropical and subtropical regions [1,2]. The transmission occurs by exposure of individuals in close proximity to wild or farm animals [3]. Recently, the disease became prevalent in cities with sanitation problems and a large population of urban rodent reservoirs, which contaminate the environment through their urine [4]. In the host, leptospirosis has a biphasic clinical presentation beginning with a septicemic followed by an immune phase with antibody production and urinary excretion of leptospires. Because of the broad spectrum of symptoms, the disease remains mostly underdiagnosed and if not treated in a proper time, the patients may develop renal damage, liver failure, and in some cases, death may occur [3,5,6]. The most severe form of leptospirosis, known as Weil’s syndrome, seen in 5 to 15% of patients, is a multisystem febrile illness, chiefly with hepatic, renal and pulmonary involvement and a mortality rate of 5 to 40% [4]. Leptospirosis presents a great economic impact since the disease affects livestock resulting in abortions, stillbirths, infertility, reduced milk production and death [3,4].

Whole-genome sequencing analysis of *L*. *interrogans* allowed identification of an array of putative leptospiral surface proteins categorized as hypothetical of unknown function [7,8]. In addition to acting as targets for the host´s immune system, it is possible that these proteins participate in initial adhesion to host cells. Indeed, many leptospiral adhesins have been identified [9]. Moreover, some adhesins are PLG-binding proteins able to generating PLA that could facilitate *Leptospira* in the host-penetration process [10,11].

In the present study, we describe the functional and immunological evaluation of two novel proteins, LIC13479 and LIC10050, identified in the genome sequences of *L*.*interrogans* serovar Copenhageni [7]. We show that these proteins are extracellular matrix (ECM) and PLG-binding proteins, which are probably expressed during infection and may participate in leptospiral pathogenesis.

## Methods

### ECM and biological components

Laminin, collagen, plasma and cellular fibronectin, elastin, vitronectin, and the control proteins fetuin and BSA were purchased from Sigma—Aldrich. (St. Louis, Mo., USA). Laminin-1 and collagen type IV were derived from the basement membrane of Engelbreth-Holm-Swarm mouse sarcoma; cellular fibronectin was derived from human foreskin fibroblasts; plasma fibronectin, vitronectin, and human complement serum were isolated from human plasma; elastin was derived from human aorta and collagen type I was isolated from rat tail. Native PLG, purified from plasma human, and factor H were purchased from EMD Chemicals, Inc. (San Diego, CA, USA). C4BP, isolated from normal human serum, was purchased from Complement Technology, INC. (Tyler, TX, USA).

### *Leptospira* strains

The pathogenic *Leptospira* strains used were *L*. *interrogans* serovar Copenhageni strain M20 (nonvirulent) and *L*. *interrogans* serovar Copenhageni strain FIOCRUZ L1-130 (virulent). The nonpathogenic *L*. *biflexa* serovar Patoc strain Patoc 1 was also used. The strains were cultured at 28°C under aerobic conditions in liquid EMJH medium (Difco, BD, Franklin Lakes, NJ) with 10% rabbit serum, enriched with L-asparagine (0.015%, w/v), sodium pyruvate (0.001%, w/v), calcium chloride (0.001%, w/v), magnesium chloride (0.001%, w/v), peptone (0.03%, w/v) and meat extract (0.02%, w/v) [12]. Bacterial cell suspensions employed in all experiments were always cultured at the same experimental conditions and harvested at log phase of growth. *Leptospira* cultures are maintained in Faculdade de Medicina Veterinária e Zootecnia, USP, Sao Paulo, SP, Brazil. For the MAT (microagglutination test), the following serovars of *Leptospira* spp. were cultured, as described above, and maintained at Instituto Adolfo Lutz, Sao Paulo, SP, Brazil: Australis, Autumnalis, Bataviae, Canicola, Castellonis, Celledoni, Copenhageni, Cynopeteri, Djasiman, Grippotyphosa, Hardjo, Hebdomadis, Icterohaemorrhagiae, Javanica, Panama, Patoc, Pomona, Pyrogenes, Sejroe, Shermani, Tarassovi and Wolffi.

### *In silico* sequence analysis

Predicted coding sequence (CDSs) LIC13479 and LIC10050 were indentified on *L*. *interrogans* serovar Copenhageni database http://bioinfo03.ibi.unicamp.br/leptospira/ [7]. CDSs selection was based on predicted cellular localization by PSORT and CELLO web servers, http://psort.hgc.jp/form.html [13] and http://cello.life.nctu.edu.tw/ [14], respectively. The SMART, http://smart.embl-heidelbergde/ [15], PFAM, http://www.sanger.ac.uk/Software/Pfam [16]and LipoP, http://www.cbs.dtu.dk/services/LipoP/ [17] web servers were used to search for predicted functional and structural domains. Conservation analyses of the coding sequences were assessed using Clustal Omega multiple-sequence alignment, http://www.ebi.ac.uk/Tools/msa/clustalo/ [18].

### Cloning, expression and purification of LIC13479 and LIC10050

The amplification of LIC13479 and LIC10050 was performed by PCR with *L*. *interrogans* serovar Copenhageni strain FIOCRUZ L1-130 genomic DNA using specific primers (Table 1). The gene sequence was amplified without the signal sequence. The PCR fragments of 1176bp (LIC13479) and 2004 bp (LIC10050) were ligated into the *E*.*coli* expression vector pAE [19] at the restriction sites presented in Table 1. Sequences were confirmed by DNA sequencing with an ABI 3100 automatic sequencer (PE Applied Biosystems, Foster city, CA). Then, plasmids pAE-LIC13479 and pAE-LIC10050 were used to transform *E*.*coli* BL21 (DE3) Star pLysS. Recombinant proteins were expressed upon addition of 1mM IPTG for 3 h under constant agitation at 37°C in the presence of 50μg/mL ampicillin and 34μg/mL chloramphenicol. The cells were harvested by centrifugation, and the resulting bacterial pellet was resuspended in lysis buffer (20mM Tris/HCL- pH8.0, 200mM NaCl, 200mg/mL lysozyme, 2 mM PMSF and 1% Triton-X114). The bacteria cells were lysed on ice with the aid of a sonication apparatus (ultrasonic processor; GE Healthcare Bio-Sciences). The insoluble fraction was recovered and resuspended in a buffer containing 20mM Tris/HCL-pH8.0, 500mM NaCl and 8M urea. The proteins were then purified through Ni^+2^- charged chelating chromatography in a Sepharose fast flow columns and dialyzed against buffer containing 500 mM NaCl and 20 mM Tris/HCL- pH 8.0 for 72 h. The efficiency of the purification and protein loss were evaluated after dialysis by 12% SDS-PAGE. Protein concentrations were estimated by comparing with predetermined concentrations of albumin (BSA—Bovine Serum Albumin).

**Table 1:** Gene locus, given names, NCBI reference sequence number, sequence of the primers used for DNA amplification and molecular mass of expressed recombinant proteins.

Gene locus^1^	Given name	NCBI reference sequence number^2^	Primer sequence (restriction site bolded)	Molecular mass (kDa)
LIC13479	Lsa46	YP_0033801	F: 5´- **CTCGAG** AGTATAAATCAAAATCCT 3´ - Xho I	46.34
			R: 5´ -**AAGCTT** CTAACGACTGATAATCTG 3´ - Hind III	
LIC10050	Lsa77	YP_0000501	F: 5´ - **CTCGAG** TCTCAACCTCTACCG 3´ - Xho I	
			R: 5´ - **AAGCTT** TCAGAGCTTTCTAAAAC 3´ - Hind III	76.67

^1^ http://aeg.ibi.ic.unicamp.br/world/lic/; LIC: *Leptospira interrogans* Copenhageni;

^2^ http://www.ncbi.nlm.nih.gov/protein/

### Circular dicrhoism (CD) spectroscopy

Purified recombinant proteins were dialyzed against sodium phosphate buffer pH 7.4 and CD spectroscopy measurements were performed at 20°C using a Jasco J-810 spectropolarimeter (Japan Spectroscopic, Tokyo) equipped with a Peltier unit for temperature control. Far-UV CD spectra were measured using a 1 mm—path—length cell at 0.5 nm intervals. The spectra were presented as an average of five scans recorded from 180 to 260 nm. The residual molar ellipticity was expressed in degree cm^2^ dmol^-1^. Spectrum data were evaluated with CAPITO software (http://capito.nmr.fli-leibniz.de/) that calculates the secondary structure content from the ellipticity experimental data [20].

### Antiserum production against Lsa46 and Lsa77

BALB/c mice (4–6 weeks old) were immunized subcutaneously with 10μg of the recombinant proteins mixed with 10% (v/v) Alhydrogel (2% Al(OH)_3,_ BrenntagBiosector) as an adjuvant. Negative control mice were injected with PBS mixed with adjuvant. Two weeks after each immunization, the mice were bled from the retro-orbital plexus, and the resulting pooled sera analysed by ELISA for the determination of antibody titres and concentration.

### Lymphoproliferation assay and cytokine production

At the end of the immunization protocols, BALB/c mice were sacrificed, their spleens were aseptically removed and cells were cultured for lymphoproliferation assay and cytokine production, essentially as described in [21].

### Immunoblotting assay

The purified recombinant proteins were loaded into 12% SDS-PAGE and transferred to nitrocellulose membranes (Hybond ECL; GE Healthcare) in a semidry equipment. Membranes were blocked with 10% non-fat dried milk in PBS containing 0.05% Tween 20 (PBS-T) and then incubated with anti-Lsa66 (1:800), Lsa77 (1: 1,500) or anti-OmpL1 (1:800) mouse polyclonal serum for 2h at room temperature. The membranes were incubated with HRP-conjugated anti-mouse IgG (1:3,000, Sigma) for 1h. Monoclonal HRP-conjugated anti-his tag antibodies (1:10,000, Sigma) were also used. The protein reactivity was revealed by a ECL reagent kit (GE Healthcare).

### Identification of LIC13479 and LIC10050 CDSs among leptospiral strains

Bacterial cultures of *Leptospira* spp. were harvested by centrifugation and washed with PBS containing 5mM MgCl_2._ After centrifugation cells were resuspended in PBS, lysed by sonication, and the resulting protein extracts were loaded into 12% SDS-PAGE and transferred to nitrocellulose membranes (Hybond ECL; GE Healthcare) in semidry equipment. Membranes were blocked with 10% non-fat dried milk in PBS containing 0.05% Tween 20 (PBS-T) and then incubated with anti-Lsa46 or Lsa77 (1: 100) mouse polyclonal serum for 2h at room temperature. Next, the membranes were incubated with HRP-conjugated anti-mouse IgG (1:3,000, Sigma). The protein reactivity was revealed by ECL reagent kit (GE Healthcare).

### Immunofluorescence assay (IFA)

The localization of LIC13479 and LIC10050 CDSs proteins by IFA was performed as follows: *L*. *interrogans* (FIOCRUZ L1-130) and *L*. *biflexa* (Patoc1), suspensions containing approximately 10^9^ cells/mL of live leptospires were harvested at 3,800 times g for 15 min, washed twice with PBS (with 50mM NaCl), resuspended in 200 μl of PBS with 2% paraformaldehyde for 40 min at 30°C under gentle shaking. After incubation, the leptospires were washed gently with PBS and incubated for 1h at 30°C (under gentle shaking) with polyclonal mouse anti-serum against LipL21, Lsa46, Lsa77, PBS and DnaK [22] at a 1:50 dilution. The leptospires were washed (PBS containing 1% BSA) and incubated with anti-mouse IgG antibodies conjugated to fluorescein isothiocyante (FITC, Sigma) at a dilution 1:50 for 50 min. Leptospires were then washed and resupended in 50μl of PBS containing 0.03μg propidium iodide (Sigma- Aldrich), 50 μl anti-fading solution (ProLong Gold, Molecular Probes) for total volume of 100 μl. The immunofluorescence—labeled leptospires were examined using a confocal LSM 510 META immunofluorescence microscope (Zeiss, Germany).

### Microscopic agglutination test (MAT)

The microscopic agglutination test was performed according to Faine et al [4]. In brief, an array of serovars of *Leptospira* spp. as antigens were employed, as previously described. A laboratory-confirmed case of leptospirosis was defined by demonstration of a four-fold microagglutination titer rise between paired serum samples. The serovar was considered to be the one with the highest dilution that could cause 50% of agglutination. MAT was considered negative when the titer was below 100.

### Reactivity of recombinant proteins with serum samples of human leptospirosis and of unrelated febrile diseases

Human IgG antibodies against Lsa46 and Lsa77 were evaluated by ELISA. Serum samples of negative and positive MAT from confirmed leptospirosis patients and of febrile unrelated diseases, were diluted 1:100 and evaluated for total IgG using peroxidase-conjugated anti-human IgG antibodies (1:3,000, Sigma, USA). Commercial healthy human sera were used as control, and cutoff values were set at three standard deviations above the mean OD_492_ of sera from control (healthy human sera).

### Binding of recombinant proteins to ECM and serum components

Protein attachment to individual macromolecules of ECM and serum components was analyzed according to previously reported procedures [23] with some modifications. In brief, ELISA plates (Costar High Binding; Corning) were coated with 1μg each component or the negative controls BSA and fetuin in 100 μl PBS for 16h at 4°C. At the next day, plates were blocked with 10% non-fat dried milk in PBS-T for 2h; thereafter 1μg of each recombinant protein was added per well allowing binding to the different components for 2h at 37°C. After washing with PBS-T, bound proteins were detected by addition of an appropriate dilution of mouse antiserum that resulted in an A_492_ value of 1 in previous titrations in 100μl PBS (1:800 for Lsa46 and 1:1,500 for Lsa77). Incubation proceeded for 1h at 37°C and after 3 washes with PBS-T, 100μl of a 1:3,000 dilution of HRP-conjugated goat anti-mouse IgG in PBS was added per well, followed by 1 h incubation at 37°C. The reactivity was detected with OPD substrate (1mg/ml) in citrate phosphate buffer (pH5.0) plus 1μl/mL H_2_O_2_ in 100 μl per well. The reaction proceeded for 10 min and was interrupted by the addition of 50 μl of 4N H_2_SO_4_. The absorbance at 492nm was determined in a microplate reader (TP- reader, Thermo). Binding was also confirmed by using HRP-conjugated anti-His mAbs previously titrated against the recombinant protein and used at a dilution that generates an A_492_ value of approximately 1.

### Dose-response curves and K_*D*_ values

ELISA plates were coated overnight with 1μg laminin, plasma fibronectin or PLG. Plates were then blocked and increasing concentrations of each purified recombinant proteins was added, ranging from 0 to 5,000 nM, depending on the component, followed by incubation for 2h at 37°C. The assessment of bound protein was performed with polyclonal antiserum raised in mice against each protein followed by HRP- conjugated anti-mouse IgG. The ELISA data, when reactions reached a saturation point, were used to calculate the equilibrium dissociation constant (K_*D*_), according to a method described elsewhere [24], following the equation K_D_ = (A_max_ [protein])/A)-[protein], where A is the absorbance at a given protein concentration, _Amax_ is the maximum absorbance for the ELISA plate reader (equilibrium), [protein] is the protein concentration and K_D_ is the equilibrium dissociation constant for a given protein concentration (ELISA data point).

### Binding characterization of recombinant proteins to PLG and PLA generation assay

To determine the role of lysine residues in PLG-recombinant protein interactions, the lysine analogue 6-aminocaproic acid (ACA) (Sigma), together with the recombinant protein at a final concentration of 2 or 20mM, was added to the PLG-coated wells. The detection of bound protein was performed as described above. For accessing the PLA generation from PLG bound to the recombinant proteins, ELISA plates were coated overnight with 10μg/mL recombinant proteins in PBS at 4°C. BSA was employed as negative control. Plates were washed with PBS-T and blocked (PBS-T 10% non-fat dry milk) for 2h at 37°C. The blocking solution was discarded and 10μg/mL human PLG was added, followed by incubation for 2h at 37°C. Wells were washed and then 4ng/well of human uPA (Sigma-Aldrich) was added. Subsequently, 100μl/well of plasmin—specific substrate _D_-valyl-leucyl-lysine-*p*-nitroanilide dihydrochloride (Sigma-Aldrich) was added at a final concentration of 0.4 mM in PBS. Plates were incubated overnight at 37°C and substrate degradation was measured by taking readings at 405 nm.

### Fibrinogen degradation assay

Lsa46 or Lsa77 (10μg/mL) was immobilized onto 96 wells plate for 16h. Plates were washed three times with PBS-T and blocked for 2h at 37°C with 3% BSA diluted in PBS. The blocking solution was discarded and PLG (20μg/mL) was added and incubated for 1h at 37°C. Wells were washed three times with PBS-T, in order to remove free PLG, and 1 μg of human purified fibrinogen (Sigma, USA) together with plasminogen activator uPA (3U) were added. Reaction mixtures were incubated for 16h at 37°C, separated by SDS-PAGE and transferred into nitrocellulose membranes. The membranes were blocked by incubating overnight at 4°C with 10% non-fat dry milk. The fibrinogen detection was performed by incubations with goat anti-human fibrinogen antibodies (1:3,000) and rabbit anti-goat secondary antibodies conjugated with HRP (1:30,000). The membranes were developed with ECL (GE Healthcare).

### Antibody inhibition assay

The effect of anti-Lsa46 and anti-Lsa77 sera on the binding of the corresponding recombinant protein to laminin, plasma fibronectin and PLG was evaluated in a dose-dependent manner. Plates were coated with 1µg each component and blocked with 10% non-fat dried milk in PBS-T. At the same time, 1µg each recombinant protein was incubated with different concentrations of the corresponding antiserum (from 1:50 to 1:400 dilutions) or anti-PBS/adjuvant (used as control) for 2 h at 37°C. Blocked recombinant proteins were then allowed to interact with the coated component for more 2 h at 37°C. After washing with PBS-T, 1:5,000 dilution of HRP-conjugated anti-His tag mAbs was added. The detection of bound proteins was performed as described previously.

### Ethics statements

All animal studies were approved by the Ethics Committee of the Instituto Butantan, Sao Paulo, SP, Brazil, under protocol number 890/12. The Committee in Animal Research in Instituto Butantan adopts the guidelines of the Brazilian College of Animal Experimentation. Confirmed- leptospirosis human serum samples were from Instituto Adolfo Lutz collection, Sao Paulo, Brazil, and were donated for research purposes. Serum samples from patients with other infectious diseases were obtained from the collections of the Laboratorio de Imunoepidemiologia, SUCEN, Sao Paulo, Brazil; Laboratorio de Protozoologia, IMT/USP, Sao Paulo, Brazil (sera from patients with Chagas’ disease) Laboratoriode Virologia, IMT/USP, Sao Paulo, Brazil (sera from patients with human immune deficiency virus [HIV] infection and dengue); and Nucleo de Estudos em Malária, SUCEN/IMT/USP, Sao Paulo, Brazil (sera from patients with malaria). The Ethics Committee for Research with Human Beings of ICB/University of Sao Paulo has deliberated that this project is exempt of ethics approval because it does not involve human manipulation.

### Statistical analysis

All results are expressed as the ±SD. Student’s paired *t*-test was used to determine the significance of differences between means and p<0.05 was considered statistically significant. Three or two independent experiments were performed, each one in triplicate.

## Results

### Bioinformatics analysis of the coding sequences

**Fig 1:**
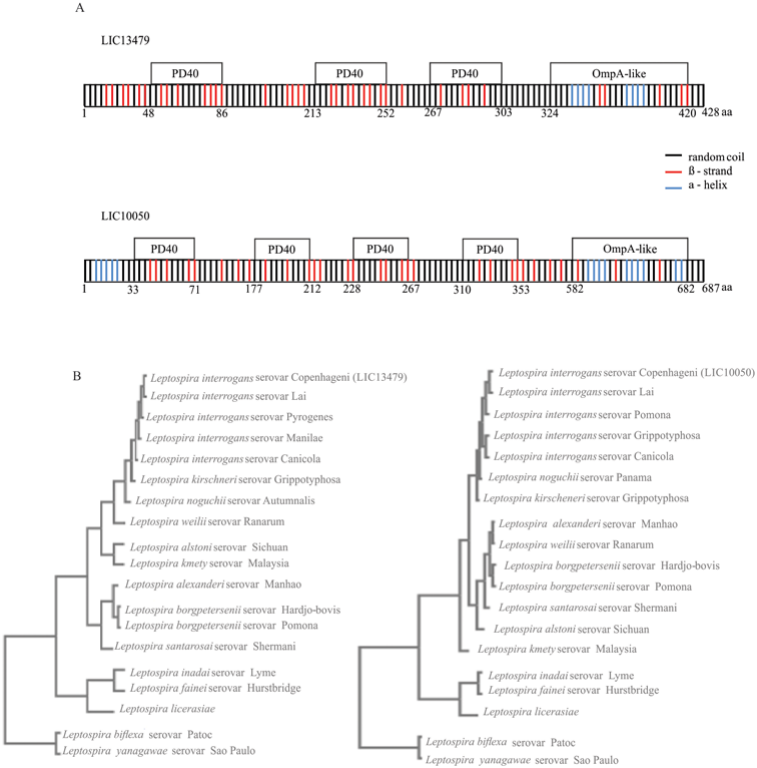
Scheme of proteins with their putative domains and sequence conservation among *Leptospira* spp. by Clustal Omega alignments. **(A)** Depicted are PD40 (from the WD40-like Beta Propeller Repeat family protein) and OmpA-like (outer membrane protein A) predicted domains identified in LIC13479 and LIC10050 CDSs by the BLAST and PFAM programs. Also shown are the regions of secondary structures, alpha helices, beta strands, and random coil structures predicted by the CAPITO program. **(B)** Blast analysis was performed among sequences of amino acids available in GenBank database and leptospiral sequences were employed to perform Clustal Omega multiple sequence alignments. The resulting phylograms show the high level of sequence conservation for LIC13479 and for LIC10050 among pathogenic strains of *Leptospira*. Intermediate and saprophyte strains show lower degree of sequence identity, and are organized in more distant branches.

The genes LIC13479, and LIC10050 were identified by analysis of the genome sequences of the chromosome I of *L*. *interrogans* serovar Copenhageni [7]. The CDSs LIC13479 and LIC10050 are predicted to be inner membrane based on PSORT [13] and outer membrane proteins by CELLO [14] programs. Putative conserved domains have been detected by BLAST and PFAM for both sequences. BLAST conserved domain predicts one and two while PFAM predicts three and four domain regions of PD40 for LIC13479 and LIC10050, respectively. OmpA_C or OmpA- like domains are found at the C-terminus regions of both CDSs. PD40 domain belongs to WD40-like Beta Propeller Repeat family protein and it is found in cell surface proteins with unknown function [25]. Proteins having the conserved OmpA-like domains are peptidoglycan-associated proteins found in several pathogens [26]. An illustration depicting LIC13479 and LIC10050 CDSs with their putative conserved domains and the regions of secondary structures predicted by the CAPITO program is shown in Fig 1A. BLAST analysis of the two CDSs showed that they are present in several strains of *Leptospira* with percentage of identity decreasing from pathogenic to intermediate to saprophyte (Table 2). Multiple sequence alignment was performed with Clustal Omega program comparing the CDSs LIC13479 and LIC10050 (Fig 1B) with the sequences available in GenBank [27]. The phylograms clearly show that the two coding sequences are well conserved and with close proximity to pathogenic strains of *Leptospira*, while the sequences present in saprophyte strains have lower similarity and are organized in a more distant branch (Fig 1B). Table 2 summarizes some features of Lsa46 and Lsa77 proteins. The CDSs, genome annotated as hypotheticals, were validated by proteomics in *L*. *interrogans* serovar Copenhageni strain FIOCRUZ L1-130, but the number of copies of each protein per cell could not be determined, probably because their amounts are below the detection limit of the method [28]. Interestingly, the protein encoded by LIC10050 is upregulated after treatment of leptospires with the antibiotics ciprofloxacin, and 162 copies per cell were detected [28], suggesting a possible role of this protein leptospiral resistance/maintenance.

**Table 2:** Gene locus, features, predicted signal peptide, protein domain and sequence conservation.

Gene locus^1^	Genome annotation	Signal peptide^2^	Domain^3^	Conservation (strain)^4^	Conservation (identity %)	References
LIC13479	Hypothetical	1–34	OmpA	*L*.*interrogans* serovar Lai	99	[29]
(Lsa46)*	protein		PD40	*L*. *santarosai* serovar Shermani	77	[30]
				*L*. *borgpetersenii* serovar	77	[31]
				Hardjo-bovis		
				*L*.*licerasiae* serovar Varillal	58	[32]
				*L*.*biflexa* serovar Patoc	45	[33]
LIC10050	Hypothetical	1–21	OmpA	*L*.*interrogans* serovar Lai	99	[29]
(Lsa77)*	protein		PD40	*L*. *santarosai* serovar Shermani	89	[30]
				*L*. *borgpetersenii* serovar	89	[31]
				Hardjo-bovis		
				*L*.*licerasiae* serovar Varillal	58	[32]
				*L*.*biflexa* serovar Patoc	44	[33]

^1^ http://aeg.ibi.ic.unicamp.br/world/lic/; LIC: *Leptospira interrogans* Copenhageni

^2^ http://www.cbs.dtu.dk/services/LipoP;

^3^ http://www.sanger.ac.uk/Software/Pfam;

^4^ http://blast.ncbi.nlm.nih.gov/Blast.cgi/

*protein given name

### Expression and purification of recombinant proteins

**Fig 2:**
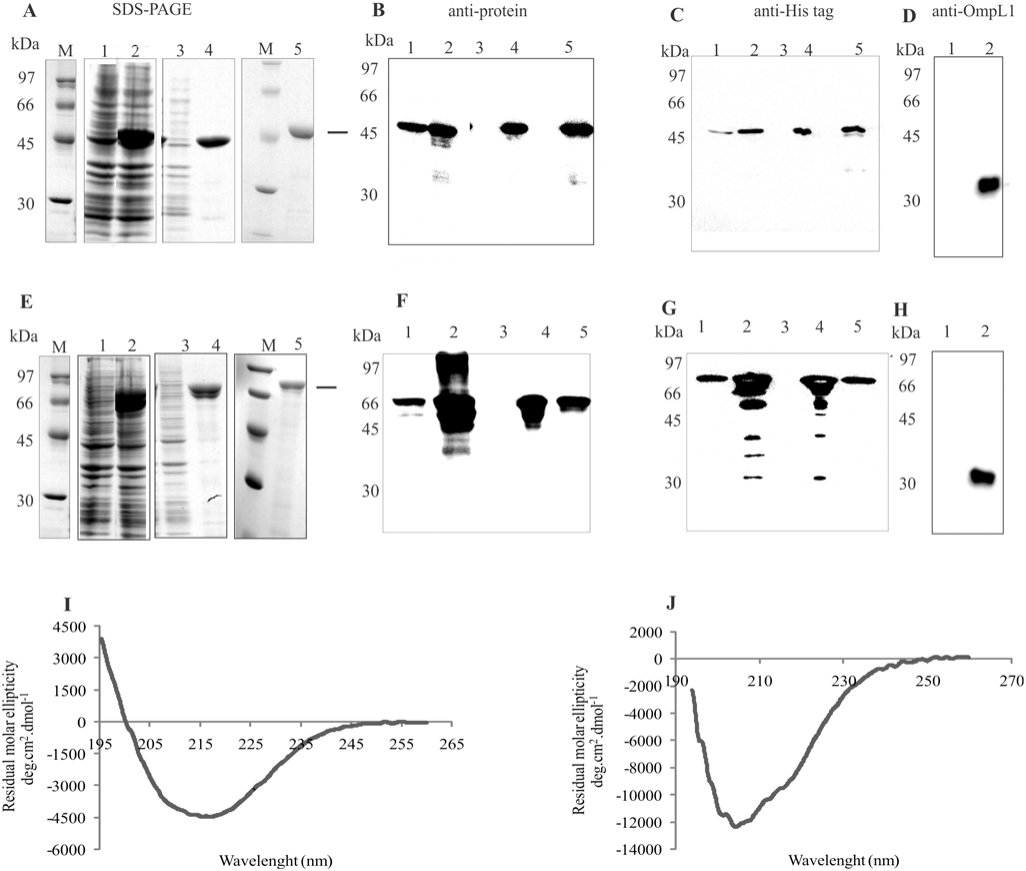
Expression, purification and Western blotting of recombinant proteins. Expression and purification analysis of recombinant proteins Lsa46 **(A)** and Lsa77 **(E)** from *E*. *coli* BL21 (DE3) Star pLysS were performed by SDS-PAGE. Lanes: molecular mass protein marker (M); non-induced total bacterial extract (1); induced total bacterial extract (2); soluble fraction (3); insoluble fraction (4); purified recombinant protein (5). Western blotting of Lsa46 or Lsa77 probed with the respective polyclonal antiserum **(B)** and **(F)** or with anti-His tag mAbs **(C)** and **(G)**, respectively. Western blotting lanes 1 to 5 refer to the same sample condition as in SDS-PAGE. Lsa46 and Lsa77 in lane 1 of **(D)** and **(H)**, respectively, and OmpL1 His-tag recombinant protein in lane 2 of **(D)** and **(H)** were probed with anti- OmpL1. Secondary structure evaluation obtained by circular dichroism spectra of the recombinant proteins: Lsa46 **(I)** and Lsa77 **(J)**. The far-UV CD spectra are presented as an average of five scans.

The selected coding sequences, without the signal peptide sequence were PCR amplified, cloned and expressed as His-tagged proteins in *E*. *coli*. Gene locus, given name, NCBI reference number, primer sequences with restriction cloning sites used for PCR amplifications and expected molecular mass of the recombinant proteins are depicted in Table 1. The recombinant proteins were purified by nickel affinity chromatography, and an aliquot of each protein was analyzed by SDS-PAGE and shown in Fig 2A and 2E, for LIC13479 (Lsa46) and LIC10050 (Lsa77), respectively. The results show that both proteins are expressed in their insoluble forms, in bacterial cell pellets, as seen in lane 4 of each Coomassie blue stained figure. Purification was successfully achieved, as shown by the presence of protein major bands in lane 5 of the same figures. Western blotting analysis of Lsa46 (Fig 2B and 2C) and Lsa77 (Fig 2F and 2G) were performed and the proteins were probed with anti-Lsa46 (Fig 2B) and anti-Lsa77 (Fig 2F) polyclonal antibodies, whereas in Fig 2C and 2G, the proteins Lsa46 and Lsa77 were detected with anti-His mAbs, respectively. In the case of Lsa46, western blotting probed with both sera detected Lsa46 as the only protein band, while for Lsa77 additional protein bands were detected. In the case of polyclonal serum, these protein bands are probably due to non-specific reaction, while for mAbs the reactivity are with lower mass protein bands, possibly caused by some Lsa77 degradation. In any event, after purification, only Lsa77 protein bands were detected (Fig 2F and 2G, lane 5). The specificity of the antibodies raised against both proteins was assessed by including OmpL1, a leptospiral His-tagged recombinant protein [21]. No reactivity was observed when OmpL1 was probed either with anti-Lsa46 or anti-Lsa77, indicating that these antibodies were not directed against His-tag (data not shown). As expected, anti-OmpL1 recognized OmpL1 but not Lsa46 and Lsa77 proteins (Fig 2D and 2H, lane 1). Structural integrity of the purified proteins was assessed by circular dichroism (CD) spectroscopy, depicted in Fig 2I for Lsa46 and Fig 2J for Lsa77, and the spectral data per wavelength analyzed by the CAPITO software [20]. The results show 10, 36 and 51% of alpha helix, beta-strands and random for Lsa46, and 21, 0.1 and 70% of alpha helix, beta-strands and random secondary structures in the case of Lsa77. Although with different percentage, a combination of secondary structures was predicted by the capito program, including random structures for both proteins, 19 and 33% for Lsa46 and Lsa77, respectively. Random secondary structure (23%) has also been found for another adhesin, LipL53 [34], but whether this structure might affect the function of these proteins is unknown and remains to be studied.

### Presence of Lsa46 and Lsa77 orthologs among virulent and saprophyte strains of *Leptospira* by IFA

**Fig 3:**
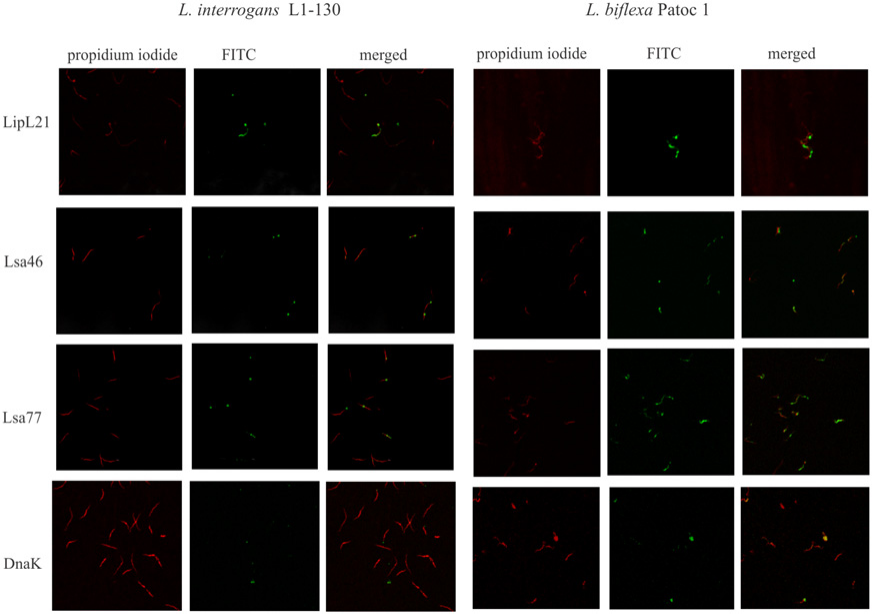
Cellular localization of native proteins in *Leptospira*. Virulent *L*. *interrogans* serovar Copenhageni strain FIOCRUZ L1-130 (left panel) and saprophyte *L*. *biflexa* serovar Patoc strain Patoc1 (right panel) were fixed with paraformaldehyde and polyclonal anti-Lsa46 and anti-Lsa77 were used to identify surface-exposed protein; serum against LipL21 and DnaK were used as a marker for surface exposed and non-exposed, cytoplasmic protein, respectively. FITC-conjugated secondary antibodies were used to reveal the surface-bound antibodies. Leptospires were identified by propidium iodide staining of the DNA. Co-localization is shown in the merged images.

In order to assess whether the chosen CDSs are located at the bacterial surface, we set out to analyze the protein location by using immunofluorescence microscopy. We also evaluated the presence of Lsa46 and Lsa77 orthologs in *L*. *biflexa* saprophyte strain. We have included LipL21, a leptospiral surface antigen, and DnaK, a cytoplasmic protein, as positive and negative controls, respectively. Leptospires were visualized by propidium iodide staining (Fig 3), followed by protein detection with the corresponding polyclonal antiserum, raised in mice against each protein, in the presence of anti-mouse IgG antibodies conjugated to FITC. Green fluorescence could be observed for LipL21, Lsa46 and Lsa77 in both strains tested, but not with DnaK, used as a negative control. These assays also confirm the presence of both proteins in *Leptospira* strains, most probably located at cell surface. The fluorescence observed with anti-Lsa46 and anti-Lsa77 seems be localized at the distal ends of the cells contrasting to the one observed with LipL21 that seems to be distributed along the bacteria. It is also possible that this visual pattern is due to low protein content of Lsa46 and Lsa77 orthologs as estimated by quantitative proteomics [28].

### Immunological evaluation of Lsa46 and Lsa77

**Fig 4:**
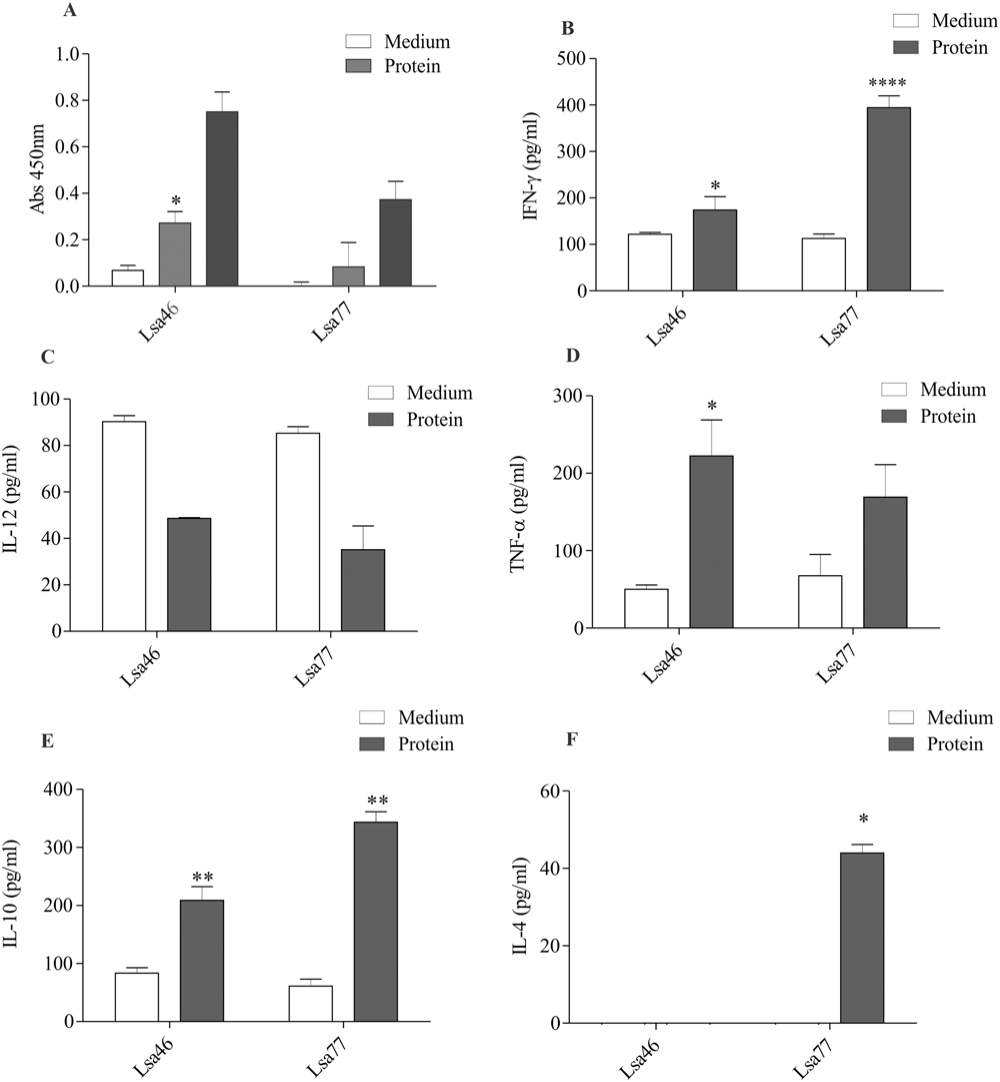
Assessment of mice immune response elicited by Lsa46 and Lsa77. **(A)** Lymphocytes proliferation in response to mice immunization with recombinant proteins. Cells were cultured and stimulated with medium alone (negative control) or recombinant proteins (5μg / ml). ConA was used as a positive control (not shown). The proliferative response was measured by a colorimetric Brdu-ELISA. Bars represent the mean absorbance at 450nm ± the standard deviation of three replicates and are representative of two independent experiments. Spleen cells were isolated and cultured by 48h in presence of medium or recombinant proteins. Cell-free supernatants were collected and the level of cytokines IFN-γ **(B)**, IL-12 **(C)**, TNF-α **(D)**, IL-10 **(E)** and IL-4 **(F)** was assayed by ELISA. For statistical analyses, comparisons were made between cells from immunized animals that received stimuli *in vitro* (gray bars) and the ones treated with medium only (white bars) by the two-tailed *t*-test (*P<0.05, **P<0.01 and ****P<0.0001).

In order to characterize the humoral and cellular immune response of these proteins, mice were immunized with Lsa46 and Lsa77. After two boosters, antibodies were measure by ELISA and titres of 20,000 and 100,000, were obtained for Lsa46 and Lsa77, respectively (data not shown). A high lymphoproliferation level was obtained when cells were treated with ConA, employed as a positive control (data not shown). The recombinant protein Lsa46 was capable to promote lymphoproliferation on cultured cells of immunized animals, whereas for immunization with Lsa77 no statistically significant value was obtained, when compared to lymphocytes from animals that had not been primed with the recombinant protein (culture medium) (Fig 4A). Supernatants of cultured spleen cells from Lsa46 and Lsa77 immunized mice were assessed for the presence of the cytokines IL-10, IL-12, IL-4, IFN-γ and TNF-α, selected to differentiate cellular Th1 (IFN-γ, TNF-α, and IL-12) and humoral Th2 (IL-10 and IL-4) immune responses. Lsa46 promoted an induction of IFN-γ, TNF-α and IL-10 cytokines (Fig 4B, 4D and 4E), with statistically significant values when compared to immunized but not stimulated animal cells. In addition to all cytokines listed for Lsa46, Lsa77 also promoted an enhancement of IL-4 (Fig 4B, 4E and 4F). Although both proteins elicited IFN-γ and TNF-α, neither promoted an increase in IL-12 level.

### Reactivity of Lsa46 and Lsa77 with human serum samples

**Fig 5:**
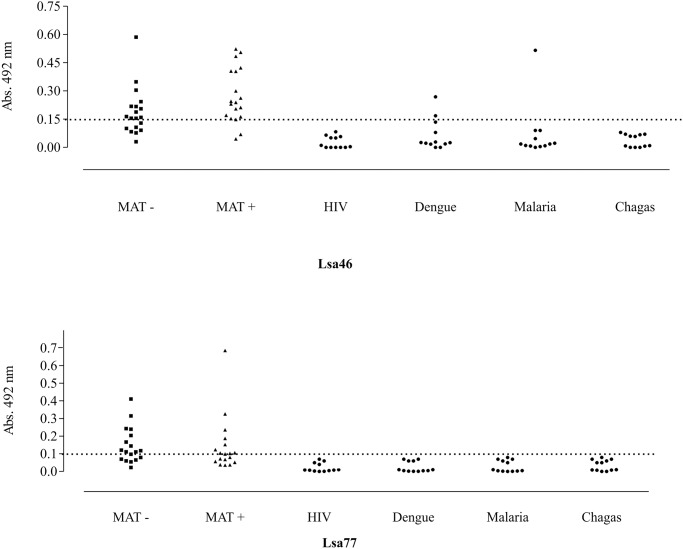
Detection of antibodies against recombinant proteins in human leptospirosis and in unrelated febrile diseases serum samples. Reactivity of recombinant proteins, Lsa46 and Lsa77 with human leptospirosis paired serum samples at the onset (MAT-) and at the convalescent (MAT+) phase, and with human serum samples from patients diagnosed with unrelated febrile diseases. The cutoff values (dashed lines) are defined as the mean plus 3 standard deviations obtained with normal human sera. *Leptospira* serum samples show 63 and 66% with MAT- and 84 and 55% with MAT+ serum samples, for Lsa46 and Lsa77, respectively.

To examine whether Lsa46 and Lsa77 are capable of inducing an immune response in infected host, we assessed the reactivity of the proteins measuring IgG antibodies present in paired serum samples at the onset (MAT-) and at the convalescent (MAT+) phase of leptospirosis. We performed an ELISA using 36- and 38-paired samples, half for each phase, for Lsa46 and Lsa77, respectively. The results depicted in Fig 5 show that both proteins are very reactive in both phases of the disease: 63 and 66% with MAT- and 84 and 55% with MAT+, for Lsa46 and Lsa77, respectively. The performances of proteins at the onset of leptospirosis, when MAT is still negative, are remarkable, suggesting that these proteins might be useful for diagnostic purposes. Due to the non-specific clinical symptoms of leptospirosis, we analyzed the reactivity of recombinant proteins Lsa46 and Lsa77 with serum samples from patients with unrelated infectious diseases that did not have a previous history of leptospirosis, including dengue (n = 12), malaria (n = 12), Chagas’ disease (n = 12), and HIV infection (n = 12). The reactivity obtained with Lsa46 and Lsa77 and these serum samples was below the cut-off obtained from human healthy donors, except that Lsa46 showed reactivity with 2 and 1 serum samples of dengue and malaria, respectively (Fig 5). The specificity of Lsa77 and Lsa46 was calculated to be 100% for all unrelated diseases tested, except that Lsa46 with dengue and malaria dengue, the specificity was calculated to be 83.3 and 91.2%, respectively.

### Binding of recombinant proteins to ECM components

**Fig 6:**
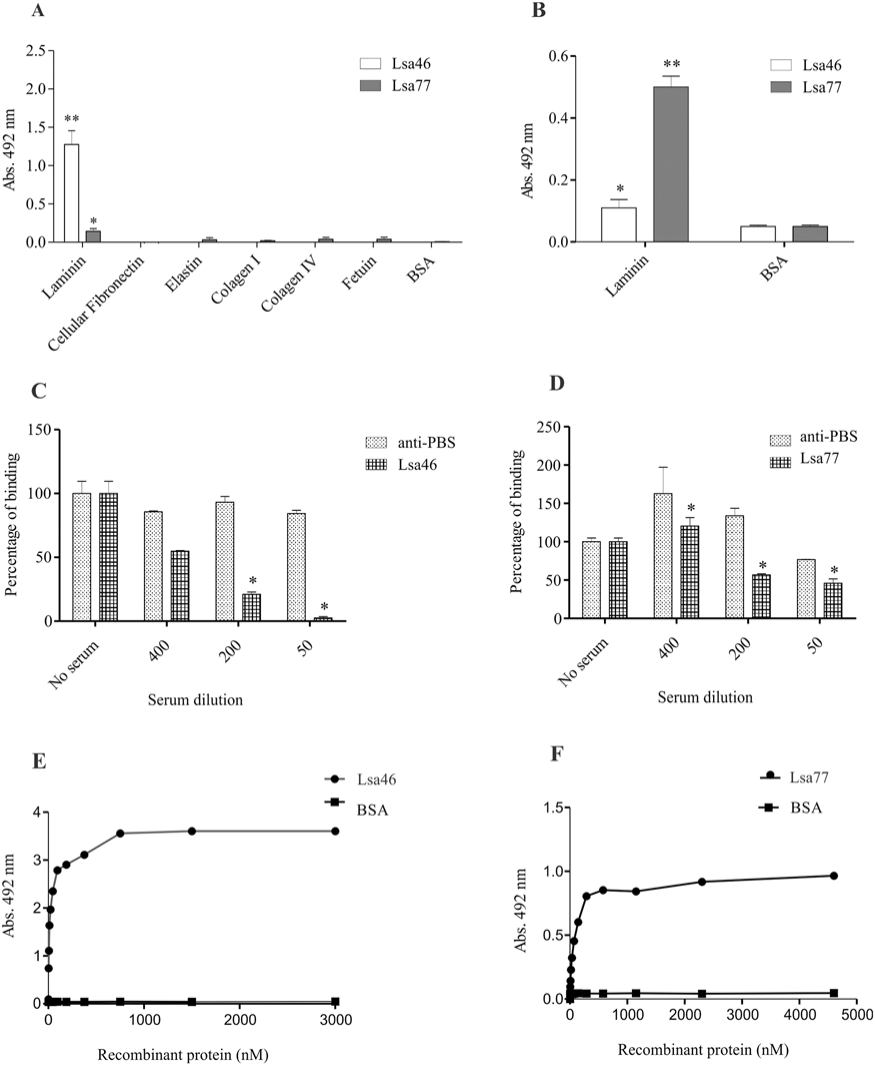
Interaction of Lsa46 and Lsa77 with ECM components. **(A)** Wells were coated with 1 μg of ECM macromolecules and the control proteins, BSA and fetuin. One μg of Lsa46 and Lsa77 were added per well and attachments were measured by ELISA. Binding to laminin was confirmed by employing anti-His mAbs **(B)**. All data represent mean ± the standard deviation from three independent experiments. For statistical analyses, the binding of recombinant proteins to the ECM components was compared to its binding to BSA by the two-tailed *t*-test (*P<0.05 and **P<0.01). **(C)** and **(D)** show the effect of mouse polyclonal anti-Lsa46 and anti-Lsa77 sera upon the binding of the corresponding protein with laminin, as compared with the binding in the absence of antibodies (*P<0.05). Serum from mice immunized with PBS and adjuvant (Alhydrogel) was used as control. Effect of increasing protein concentration on the binding to a constant laminin concentration: Lsa46 **(E)** and Lsa77 **(F)**. Each point represents values determined in triplicate and data are expressed as the mean absorbance value at 492nm. BSA was used as a negative control. The dissociation constants (K_*D*_) were calculated based on ELISA data for the recombinant protein that reached equilibrium: 24.3± 17.0 nM and 53 ±17.5 nM, for Lsa46 and Lsa77, respectively.

The Lsa46 and Lsa77 proteins are suggested by bioinformatics and immunofluorescence microscopy to be surface-exposed. We thus set out to evaluate whether these proteins could mediate host colonization by binding to extracellular matrix proteins. Hence, laminin, collagen Type I, collagen Type IV, cellular fibronectin, elastin and the control proteins fetuin and BSA were immobilized on microdilution plates and recombinant protein binding was assessed by an ELISA using polyclonal antibodies against each of the protein (Fig 6A) and anti-His mAbs (Fig 6B). Lsa46 and Lsa77 proteins were reactive to laminin, whereas no statistically significant binding capacity was observed with both proteins when the wells were coated with collagen I and IV, elastin, or with the highly glycosylated control protein fetuin and BSA (Fig 6A). The interactions of Lsa46 and Lsa77 with laminin were confirmed when the reaction was detected with anti-His mAbs antibodies (Fig 6B). The attachment of Lsa46 to laminin was inhibited when the protein was previously treated with anti-Lsa46 prior to addition of ECM component. The inhibition was dependent on serum dilution and binding was abolished at 1:50 dilution (Fig 6C). Similar experiment performed with Lsa77 and laminin showed that the reaction was inhibited by anti-Lsa77, but in this case, only 50% inhibition was achieved at the lowest serum dilution employed (Fig 6D). The data suggest that, contrary to Lsa46, the interaction of Lsa77 with laminin involves other regions in addition to the immunogenic epitopes. Metaperiodate oxidation of laminin caused no significant reduction in the binding activity, of Lsa46 and Lsa77, suggesting that laminin carbohydrate moieties are not important for these interactions (not shown). These data are similar to those obtained with OmpL1 [21] but differ from LipL53 and Lsa25 laminin-binding proteins [34,35], implying that leptospiral adhesins do not all interact with laminin at the same site. To evaluate whether the interactions of recombinant proteins to laminin fulfill the properties of a typical receptor-ligand binding, we performed ELISA to determine dose-response curves, increasing the protein concentration while keeping the ligand concentration constant. Dose-dependent and saturable curves were observed with Lsa46 (Fig 6E) and Lsa77 (Fig 6F). Binding saturation level was reached by Lsa46 and Lsa77 at protein concentration of 1,500 and 3,000 nM, respectively. The calculated dissociation equilibrium constants (*K*_D_) for the recombinant proteins Lsa46 and Lsa77 were: 24.3±17.0 nM and 53 ±17.5 nM, respectively. Table 3 compares the affinities for the multiple ligands of the various proteins characterized in this laboratory.

**Table 3:** Comparison of the binding affinities for the multiple ligands of the proteins characterized by this group.

LIC*	Given Name	Ligands	K_*D*_(nM)	Effect of metaperiodate	Effect of ACA	Plasmin generation	Refs
LIC13479	Lsa46	Laminin	24.3±17	No	-	-	**
		Plasminogen	26.3±11.7	-	Yes	Yes	
		Plasmatic	ND	-	-	-	
		Fibronectin					
LIC10050	Lsa77	Laminin	53±17.5	No	-	-	**
		Plasminogen	ND	-	Yes	Yes	
		Plasmatic	ND	-	-	-	
		Fibronectin					
LIC12906	Lsa24/	Laminin	ND	Yes	-	-	[36]
	LenA	Plasminogen	ND	-	Yes	Yes	[37]
LIC12690	Lp95	Laminin	ND	ND	-	-	[38]
		Fibronectin	ND		-	-	
LIC10368	Lsa21	Laminin	ND	Yes	-	-	[23]
		Collagen IV	ND	-	-	-	
		Fibronectin	ND	-	-	-	
LIC10314	Lsa63	Laminin	ND	ND	-	-	[39]
	Collagen IV	ND	-	-	-		
LIC12099	LipL53	Laminin	ND	Yes	-	-	[34]
		Collagen IV	ND	-	-	-	
		Fibronectin	ND	-	-	-	
LIC10258	Lsa66	Laminin	55±15	ND	-	-	[40]
		Fibronectin	290±11	-	-	-	
		Plasminogen	68.8±25.2	-	ND	Yes	
LIC12880	Lp30	Plasminogen	167±60	-	ND	Yes	[40]
LIC11087	Lsa30	Laminin	292±24	ND	-	-	[41]
		Fibronectin	157±35	-	-	-	
		Plasminogen	ND	-	ND	Yes	
		C4BP	ND	-	-	-	
LIC10973	OmpL1	Laminin	2099±871	ND	-	-	[21]
		Fibronectin	1239±506	-	-	-	
		Plasminogen	368±121	-	Yes	Yes	
LIC11469	Lsa20	Laminin	1988±563	ND	-	-	[42]
		Plasminogen	509±77	-	ND	Yes	
LIC12253	Lsa25	Laminin	415±203	Yes	-	-	[35]
		C4BP	ND	-	-	-	
LIC11834	Lsa33	Laminin	367±248	ND	-	-	[35]
		C4BP	ND	-	-	-	
		Plasminogen	23.5±4.66	-	ND	Yes	
LIC11360	Lsa23	Laminin	25.1±4.1	ND	-	-	[43]
		Plasminogen	11.7±1.4	-	Yes	Yes	
		Fibronectin	528±143	-	-	-	
		Fator H	ND	-	-	-	
		C4BP	17.6±3.5	-	-	-	
LIC11009	Lsa26	Laminin	952±418	ND	-	-	[43]
		Plasminogen	6.4±2.5	-	Yes	Yes	
LIC11975	Lsa36	Laminin	120±97.6	ND	-	-	[43]
		Plasminogen	17.8±5.5	-	Yes	Yes	
		Fibronectin	34.8±10.4	-	-	-	
LIC10645	Lsa44	Laminin	108±43	ND	-	-	[44]
		Plasminogen	53.5±18.4	-	Yes	Yes	
LIC10731	Lsa45	Laminin	250.38	ND	-	-	[44]
		Plasminogen	36.8±20.3	-	Yes	Yes	
LIC10829	Lsa32	Laminin	ND	ND	-	-	[45]
		Plasminogen	81.5±31.1	-	Yes	Yes	

*Leptospira interrogans serovar Copenhageni L1-130 genome annotation;(http://aeg.lbi.ic.unicamp.br/world/lic/; Nacimento et al., 2004);

** proteins in this study;

ND, not determined; K_*D*_, dissociation constant.

### Binding of Lsa46 and Lsa77 to human plasma components

**Fig 7:**
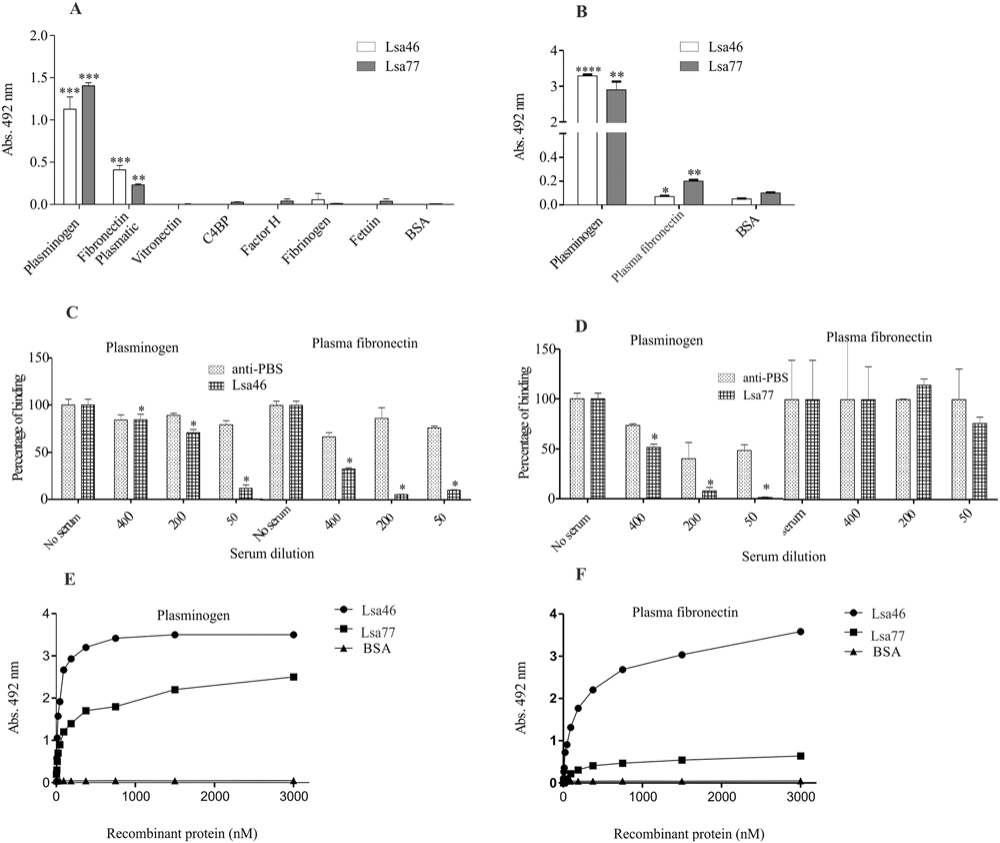
Assessment of Lsa46 and Lsa77 binding with plasma components. **(A)** Wells were coated with 1 μg of each plasma components and the control proteins, BSA and fetuin. One μg of Lsa46 and Lsa77 were added per well and binding was measured by ELISA. Binding to plasminogen and plasma fibronectin was confirmed by employing anti-His mAbs **(B)**. All data represent mean ± the standard deviation from three independent experiments. For statistical analyses, the binding of recombinant proteins to the plasma components was compared to its binding to BSA by using two-tailed *t*-test (*P<0.05, **P<0.01 and ****P<0.0001). Effect of mouse polyclonal anti-Lsa46 **(C)** and anti-Lsa77 **(D)** serum upon the binding of the corresponding protein with plasminogen and plasma fibronectin was compared with the binding in the absence of antibodies (*P<0.05). Immunized mice serum with PBS in Alhydrogel association was used as control. Dose-dependent binding of Lsa46 **(E)** and Lsa77 **(F)** to plasminogen and plasma fibronectin was performed. Each point represents values determined in triplicate and data are expressed as the mean absorbance value at 492nm. BSA was used as a negative control. The dissociation constant (K_*D*_) was calculated based on ELISA data for Lsa46 that reached equilibrium, as 26.2 ± 11.7 nM.

We have previously shown that *Leptospira* bind PLG and that several proteins, including some adhesins, could act as binding proteins at the bacterial surface [10,46]. Hence, we decided to evaluate whether Lsa46 and Lsa77 were also capable of binding human PLG *in vitro*. We have also assayed other plasma components: plasma fibronectin, vitronectin, C4BP, factor H, and fibrinogen. Components were individually coated onto ELISA plates and allowed to interact with the recombinant proteins Lsa46 and Lsa77. The results show that Lsa46 and Lsa77 attach to PLG and to plasma fibronectin, when the reaction was probed with polyclonal antibodies against each protein (Fig 7A). The data were confirmed when the bindings were detected with anti-His mAbs (Fig 7B). No reactivity was detected with the other plasma components tested. We also investigate whether immune epitopes were involved in the binding of both proteins with PLG and plasma fibronectin, by pre-incubating the proteins with the respective antibody. The results demonstrate that immune epitopes are involved in the binding of both proteins with PLG (Fig 7C and 7D) and of Lsa46 with plasma fibronectin (Fig 7C). Almost no inhibition on the binding of Lsa77 and plasma fibronectin was achieved when the protein was incubated with its antiserum, even at the lowest dilution employed (Fig 7D), suggesting that antibody binding regions do not participate in the interaction with this component.

The interactions between the recombinant proteins with PLG and plasma fibronectin were evaluated on a quantitative basis as depicted in Fig 7E and Fig 7F, respectively. Binding was dose-dependent when increasing concentrations of the recombinant protein Lsa46 and Lsa77 (0 to 3,000 nM) were added to constant amount of PLG (Fig 7E) and plasma fibronectin (Fig 7F). Binding saturation was reached only with Lsa46 and PLG at the protein concentration of 1,500 nM, with a dissociation equilibrium constant (*K*_D_) of 26.2± 11.7 nM (Table 3).

### Binding of Lsa46 and Lsa77 with PLG occur via lysine residues and generate PLA

**Fig 8:**
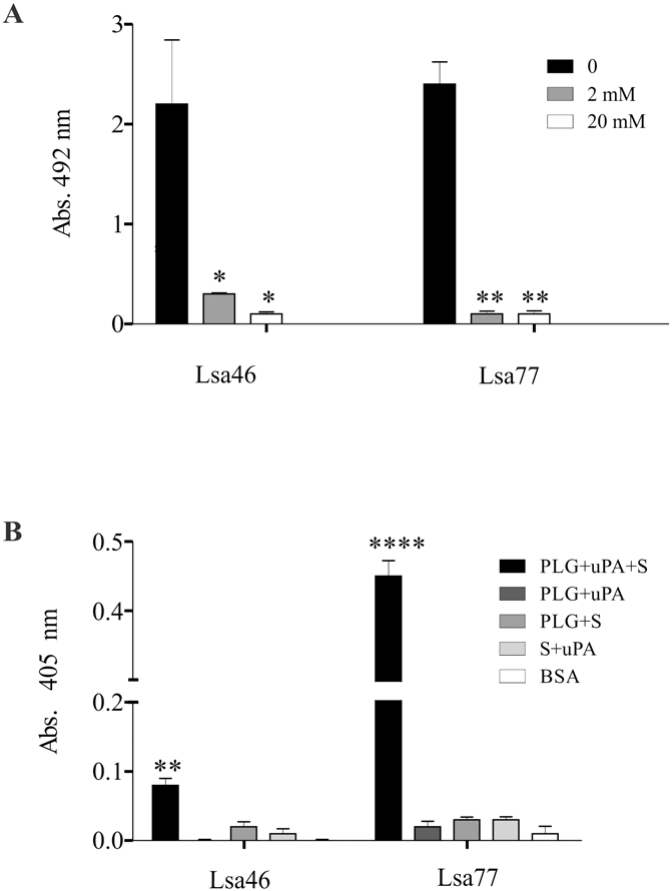
The effect of lysine analogue (ACA) on the binding of Lsa46 and Lsa77 to PLG and evaluation of PLA generation in the presence of PLG activator. **(A)** Role of lysine residues in protein-PLG interaction was assessed by ELISA. BSA was used as a negative control. Bars represent the mean absorbance at 492nm ± the standard deviation of three replicates and a representative of two independent experiments is depicted. For statistical analyses, the binding of the Lsa46 and Lsa77 in the presence of ACA was compared with the binding to PLG without ACA by the two-tailed *t*-test (*P<0.05 and **P<0.01). **(B)** PLA generation by PLG bound to Lsa46 and Lsa77 was measured indirectly by the cleavage of the specific PLA-specific substrate, _D_-valyl-leucyl-lysine-*p*-nitroanilide dihydrochloride, when the recombinant proteins were treated with PLG+uPA+S or in the absence of one of the three components (PLG+uPA/ PLG+S/ uPA+S); BSA was employed as a control protein. Bars represent mean absorbance at 405nm as a measure of relative substrate cleavage ± the standard deviation of three replicates; one representative, of two independent experiments, is shown. Statistically significant differences were observed relative to control BSA (**P<0.01 and ****P<0.0001).

PLG kringle domains frequently mediate interactions with lysine residues of the bacterial receptors [47]. The involvement of these domains was shown to contribute in the binding of PLG with *L*. *interrogans* serovar Copenhageni strain FIOCRUZ L1–130, because ACA, an analogue of lysine, profusely inhibited the binding [46]. Based on these results, we decided to investigate if lysine residues are involved in the binding of recombinant proteins with PLG, by the adding ACA to the reaction mixtures. The results strongly suggest that this is indeed the case for both Lsa46 and Lsa77 proteins, because the minimum ACA used in these assays nearly completely abolished the binding to PLG (Fig 8A).

Previous work of our group has reported that PLG bound to leptospiral binding proteins can be activated to PLA by activators [21,35,41,43,44]. To assess whether PLG attached to Lsa46 and Lsa77 proteins can also achieve proteolytic activity, as reported for several leptospiral proteins (see Table 3), microplates individually coated with the recombinant proteins were incubated with PLG. The uPA-type PLG activator was added together with a plasmin-specific chromogenic substrate (described in method section). The plasmin activity was indirectly evaluated by measuring the cleavage of the PLA-specific chromogenic substrate at 405 nm. The data show that only the complete system, Lsa46 or Lsa77, PLG, uPA and PLA substrate, can generate the expected PLA-derived product (Fig 8B). PLG bound to the proteins can be activated to PLA, via proteolytic cleavage through activators such as uPA, but Lsa77 seems to be more efficient, as seen by the higher amount of PLA generation when compared with Lsa46. BSA, which does not bind PLG, was employed as negative control, and did not show any proteolytic activity. No cleavage of the chromogenic substrate was observed in controls omitting PLG, uPA or the chromogenic substrate, respectively. However, when PLG/PLA is bound to recombinant proteins no fibrinogen degradation products were detected (not shown).

## Discussion

The characterization of leptospiral outer membrane proteins is critical to understanding leptospirosis pathogenicity. The OmpA-like domains (named after the description of C-terminal domain of *Escherichia coli* OmpA protein) have been shown to be non-covalently associated with peptidoglycan [48]. Proteins having OmpA-like domains of important pathogens were described to be involved in different aspects of infection. OmpA outer membrane protein of *Escherichia coli* has been reported to act as adhesin/invasin and to participate in biofilm formation [49]. Other OmpA-like proteins include *E*. *coli* lipoprotein PAL (Peptidoglycan-associated lipoprotein) [50], the *Neisseria meningitides* Rmp [51], and the peptidoglycan-associated lipoprotein (Pal) of *Haemophilus influenza* that is been considered a potential vaccine candidate against this bacteria [52]. The Loa22 was the first OmpA-like protein described in *Leptospira* and shown to be reactive with convalescent mouse sera. [53]. By mutagenesis experiments, this protein was reported to be essential for leptospiral virulence [54]. The OmpA-like proteins Omp52 and OmpA70 have been described for *L*. *santarosai* serovar Shermani and *L*. *interrogans* serovar Copenhageni, respectively [55,56]. The first protein is environmentally regulated and expressed in human confirmed leptospirosis patients, while the latter has been shown to be highly immunogenic in mice.

Although whole genome sequences of several *Leptospira* have created unquestionable contribution to understanding host-pathogen interactions, mechanisms driven by the bacteria during infection remain to be elucidated. We have been exploring the genome sequences of *L*. *interrogans* serovar Copenhageni searching for proteins annotated as hypothetical and surface-exposed. Through these criteria, we have identified and characterized several adhesins, including one with OmpA-like domain [9,40], proteins that interact with PLG [10,21,35,40,41,42,44,57], proteins that bind regulators of complement system [35,43] and proteins recognized by antibodies present in sera of human patients infected with leptospires [21,39,42,43,44,58].

In this work, we report the characterization of two novel hypothetical proteins having an OmpA-like domain at C-terminus, both of which are surface-exposed leptospiral adhesins, called Lsa46 and Lsa77. These proteins, encoded by the genes LIC13479 and LIC10050, were expressed in *E*. *coli*, as 46 and 77 kDa recombinant proteins, respectively.

The protein sequences are well preserved among pathogenic species of *Leptospira*, whereas lower identities were found in intermediate and saprophyte strains. We confirm the expression of both proteins in low-passage virulent and saprophyte strains of *Leptospira*. The expression of proteins was not detected by immunoblotting in saprophyte strains (data not shown), probably due the lower sensitivity of the method when related to fluorescence. By comparing with the fluorescence of LipL21, a surface lipoprotein of *Leptospira* [59], the proteins are most probably surface exposed.

Lsa46 and Lsa77 proteins exhibit extracellular matrix-binding properties. It is thus possible that they may play a role in the attachment to host tissues. To date, several leptospiral ECM-binding proteins have been reported [9,21,23,34,35,38,42,43,60,61]. Some adhesins also bind to the complement regulators, factor H and/or C4BP, and may contribute to serum resistance of pathogenic leptospires, via complement-mediated killing [35,43,62]. Lsa46 and Lsa77 proteins exhibit a broad spectrum binding profile since it interacts with laminin, and plasma fibronectin. Likewise, other leptospiral adhesins have been reported to bind to different ECM macromolecules [63,64,65,66]. The binding affinities calculated for Lsa46 and Lsa77 with laminin are of the same order of magnitude as the values obtained for Lsa66 [40] and Lsa23 [43]. Some important pathogens such as, *Staphylococcus aureus*, *Yersinia enterocolitica* and *Haemophilus influenza*, have surface proteins that interact with diverse ECM molecules [67,68,69]. Consistent with their roles in bacterial adhesion, the corresponding Lsa46 and Lsa77 proteins in *Leptospira* are localized at the cell surface as tested by immunofluorescence assay, their capacity to induce immune response in mice and their reactivity with confirmed leptospirosis serum samples.

The interaction between pathogens and the host fibrinolytic system has been shown for several pathogens including, invasive gram-positive, gram-negative bacteria, virus and parasites [47,70,71,72,73]. Interactions with the fibrinolytic system by *Borrelia* spp. and *Treponema denticola* were suggested to have an important role during infection [74,75]. Our group have reported for the first time that *Leptospira* species were also capable to bind PLG and generating PLA, in the presence of activator[46]. To date, several leptospiral proteins have been described as PLG-binding [57] and some of them also functioning as ECM-interacting proteins [35,43,44]. We have previously reported an adhesin and PLG-binding protein with OmpA-like domain, named Lsa66 [40]. We have now identified Lsa46 and Lsa77, as novel PLG-binding proteins. The binding affinity was achieved only for Lsa46, and is of the same order of magnitude of the values calculated for other recombinant proteins reported from our laboratory [10]. Bound PLG to both proteins could be converted to plasmin by the addition of PLG activator (uPA), with specific proteolytic activity. Although we have previously shown that PLG activated to PLA on leptospiral surface is able to degrade laminin, fibronectin and fibrinogen [46,76], we did not detected fibrinogen degradation products when PLG/PLA is generated bound to recombinant proteins. One possible explanation is the number of PLG binding proteins on leptospiral surface, 17 identified to this point [10] compared to one individual protein. Another possibility is that micro-environmental settings within the bacteria may provide better reaction conditions when compared to bacterial-free reaction medium.

The binding ability of Lsa46 and Lsa77 to host-derived molecules is different. Immunogenic epitopes seem to be involved on the interaction of Lsa46 with ECM and PLG, while with Lsa77 only the binding to PLG involved these sites. Interaction with laminin and this protein was partially prevented by anti-Lsa77. Though unexpectedly, the data suggest that with Lsa77 other non-immunogenic regions are involved on the interaction with laminin and plasma fibronectin. Similar data have been reported for the recombinant OmpL37 of *L*. *interrogans*. The antiserum against this protein did not exhibit any statistically significant effect on the binding of OmpL37 to fibronectin, fibrinogen and laminin [77].

Lsa46 and Lsa77 are immunogenic, capable of eliciting Th1 and Th2 immune responses in mice. These proteins have in common, with previously described Loa22 and Lsa66 adhesins having OmpA-like domains, positive reactivity with serum samples from patients diagnosed with leptospirosis and are probably expressed during the disease [39,40,53,64,77,78,79]. Most interestingly, both proteins have higher sensitivity to detect leptospirosis at the onset of the disease than the standard reference test MAT [4], and could be further explored for early diagnosis purposes. Moreover, both proteins showed high specificity among unrelated infections diseases, commonly found in tropical countries.

In conclusion, we report in this work two novel OmpA-like proteins, Lsa46 and Lsa77, which can act as PLG-binding proteins. PLA can be generated at the leptospiral surface, endowing the bacteria with proteolytic power that could help them to overcome tissue barriers. In addition, Lsa46 and Lsa77 are ECM-binding proteins that react with antibodies present in both phases of the disease. Based on the results *in vitro* presented here, we may hypothesize that these multifunctional proteins have the potential of promoting attachment/colonization and of contributing to invasion/dissemination processes within the hosts. Cell-based assays and mutagenesis should be employed in order to gain insights on the biological role of these proteins. Moreover, we plan to investigate the protective immunity provided by of these proteins against lethal infection in the hamster model of leptospirosis. Investigating *Leptospira*–host interactions at a molecular level should enhance our understanding of aspects of pathogenesis and may help prevent and control leptospirosis.
